# Correction: Enhancement of CO_2_ binding and mechanical properties upon diamine functionalization of M_2_(dobpdc) metal–organic frameworks†
†Electronic supplementary information (ESI) available. See DOI: 10.1039/c7sc05217k


**DOI:** 10.1039/c9sc90145k

**Published:** 2019-07-01

**Authors:** Jung-Hoon Lee, Rebecca L. Siegelman, Lorenzo Maserati, Tonatiuh Rangel, Brett A. Helms, Jeffrey R. Long, Jeffrey B. Neaton

**Affiliations:** a Molecular Foundry , Lawrence Berkeley National Laboratory , Berkeley , California 94720 , USA . Email: jbneaton@lbl.gov; b Department of Physics , University of California , Berkeley , California 94720 , USA; c Department of Chemistry , University of California , Berkeley , California 94720 , USA; d Materials Sciences Division , Lawrence Berkeley National Laboratory , Berkeley , California 94720 , USA; e Department of Chemical and Biomolecular Engineering , University of California , Berkeley , California 94720 , USA; f Kavli Energy Nanosciences Institute at Berkeley , Berkeley , California 94720 , USA

## Abstract

Correction for ‘Enhancement of CO_2_ binding and mechanical properties upon diamine functionalization of M_2_(dobpdc) metal–organic frameworks’ by Jung-Hoon Lee *et al.*, *Chem. Sci.*, 2018, **9**, 5197–5206.



## 

Regrettably, in the original manuscript, an error was made in the calculations of the zero-point energy (ZPE) and thermal energy (TE) of gas-phase CO_2_. After evaluating eqn (9)–(13) in the ESI,† the authors found that the computed ZPE and TE corrections were in error by around 6.4 kJ mol^–1^ and 1.6 kJ mol^–1^, respectively. These ZPE and TE contributions alter the predicted CO_2_ binding enthalpies (*H*_B_) in Table 2. Please see below an updated Table 2, which includes the updated values for the ZPE and TE corrections and the CO_2_ binding enthalpies (*H*_B_).

**Table 2:** A comparison of computed CO_2_ binding energies (*E*_B_) and enthalpies (*H*_B_) (in kJ mol^–1^) in mmen–M_2_(dobpdc) (M = Mg, Mn, Fe, Co, Zn) with the experimental values at a CO_2_ loading of 2 mmol g^–1^.^37^ Zero-point energy (ZPE) and thermal energy (TE) corrections of ammonium carbamate and mmen are considered. All ZPE and TE values are computed at 298 K

	This work				Exp
	*E*_B_	ZPE	TE	*H*_B_	*H*_B_
Mg	74.7	–9.2	2.7	68.1	71
Mn	68.9	–8.6	2.2	62.5	67
Fe	56.2	–8.3	2.3	50.3	58
Co	52.4	–7.7	2.0	46.8	52
Zn	62.4	–7.9	2.8	57.3	57

The conclusions in the original manuscript remain unchanged upon consideration of these modified corrections, and the computed CO_2_ binding enthalpies still compare quite well with experiments, within 8 kJ mol^–1^ in the worst case (Fe) but typically better.

The Royal Society of Chemistry apologises for these errors and any consequent inconvenience to authors and readers.

